# ID 189 - Acesso a Produtos à Base de Canabidiol por Pacientes com Epilepsia Refratária Atendidos no Sistema Único de Saúde do Distrito Federal, Brasil

**DOI:** 10.5327/2237-9622.2025.v34s1.189

**Published:** 2025-11-25

**Authors:** Mariana Dias Lula, Ronaldo Portela, Daniel Marques Mota, Evandro Augusto Rubert Roza, Maria Laura Dias Alves e Silva, Augusto Afonso Guerra, Cristina Mariano Ruas

**Keywords:** canabidiol, epilepsia resistente a medicamentos, Sistema Único de Saúde

## Abstract

**Introdução:**

Nos últimos anos, o canabidiol (CBD) tem se destacado como uma alternativa terapêutica relevante para pacientes com epilepsia refratária (ER). No Brasil, embora alguns governos locais forneçam produtos à base de CBD pelo Sistema Único de Saúde (SUS) para o manejo da ER, ainda existem barreiras que dificultam o acesso e o uso adequado. A realização de avaliações da tecnologia em um contexto real de utilização é importante para compreender o acesso e a efetividade das tecnologias ofertadas. Nesse sentido, o objetivo deste estudo é avaliar o acesso aos produtos à base de canabidiol por pacientes com epilepsia refratária atendidos pela Secretaria de Saúde do Distrito Federal (SES/DF).

**Método:**

Trata-se de um estudo transversal, parte do Projeto Canabidiol – SUS, que avalia aspectos farmacoepidemiológicos, farmacoeconômicos e farmacogenômicos do CBD no âmbito do SUS. Foram incluídos pacientes com ER que se cadastraram para receber produtos à base de CBD pela SES/DF, entre 2017 e 2022. Dados sociodemográficos, clínicos e relacionados ao tratamento com CBD foram obtidos por meio documental e por meio de entrevistas telefônicas com o próprio paciente ou familiar após aprovação ética. O acesso foi avaliado a partir da pergunta "Desde o início do tratamento com canabidiol, qual(is) foi(ram) a(s) forma(s) de obtenção do produto?" Análises descritivas foram realizadas utilizando o software R versão 4.4.1.

**Resultados:**

Compuseram o estudo 101 indivíduos, sendo que 58 (57,4%) eram do sexo masculino, com idade média de 19,8 anos (DP=16,0). Do total de solicitações de CBD, 81 (80,2%) foram autorizadas para dispensação pela SES-DF. Entre os pacientes, 84 (83,2%) haviam sido tratados previamente com pelo menos dois medicamentos antiepilépticos antes da solicitação do CBD à SES/DF, com média de 3,9 antiepilépticos prévios por paciente (DP=2,1). Do total, 59 (58,4%) dos pacientes relataram nunca terem acessado o produto por meio da SES-DF. Entre os 91 indivíduos que já haviam utilizado o CBD em algum momento da vida, 63 (69,2%) relataram ter adquirido o produto com recursos próprios, 42 (46,1%) relataram ter recebido o produto via SES-DF e 29 (31,9%) relataram ter obtido o produto via judicialização. Entre os dez participantes que nunca utilizaram o CBD, nove (90,0%) declararam que o principal motivo para a não utilização foi a falta de acesso ao produto no SUS; e, entre os 51 indivíduos que interromperam o uso do CBD, 34 (66,7%) justificaram a interrupção por desabastecimento do produto na SES-DF e/ou insuficiência de recursos financeiros próprios para a aquisição.

**Conclusão:**

As dificuldades de acesso ao CBD, caracterizadas por fornecimento irregular e desabastecimento, podem comprometer a adesão ao tratamento, estar associadas a piores resultados clínicos, além de impactar a organização da assistência terapêutica no SUS. Estudos adicionais para avaliar a efetividade do CBD em condições reais de uso e a viabilidade de incorporação no SUS em âmbito nacional, são imprescindíveis para a continuidade da oferta destes produtos.

